# Luteinizing Hormone Secretion during Gonadotropin-Releasing Hormone Stimulation Tests in Obese Girls with Central Precocious Puberty

**DOI:** 10.4274/jcrpe.3091

**Published:** 2016-12-01

**Authors:** Hae Sang Lee, Jong Seo Yoon, Jin Soon Hwang

**Affiliations:** 1 Ajou University School of Medicine, Ajou University Hospital, Department of Pediatrics, Suwon, Korea

**Keywords:** luteinizing hormone, body mass index, Precocious puberty

## Abstract

**Objective:**

Girls with precocious puberty have high luteinizing hormone (LH) levels and advanced bone age. Obese children enter puberty at earlier ages than do non-obese children. We analyzed the effects of obesity on LH secretion during gonadotropin-releasing hormone (GnRH) tests in girls with precocious puberty.

**Methods:**

A total of 981 subjects with idiopathic precocious puberty who had undergone a GnRH stimulation testing between 2008 and 2014 were included in the study. Subjects were divided into three groups based on body mass index (BMI). Auxological data and gonadotropin levels after the GnRH stimulation test were compared.

**Results:**

In Tanner stage 2 girls, peak stimulated LH levels on GnRH test were 11.9±7.5, 10.4±6.4, and 9.1±6.1 IU/L among normal-weight, overweight, and obese subjects, respectively (p=0.035 for all comparisons). In Tanner stage 3 girls, peak stimulated LH levels were 14.9±10.9, 12.8±7.9, and 9.6±6.0 IU/L, respectively (p=0.022 for all comparisons). However, in Tanner stage 4 girls, peak stimulated LH levels were not significantly different among normal, overweight, and obese children. On multivariate analysis, BMI standard deviation score was significantly and negatively associated with peak LH (β=-1.178, p=0.001).

**Conclusion:**

In girls with central precocious puberty, increased BMI was associated with slightly lower peak stimulated LH levels at early pubertal stages (Tanner stages 2 and 3). This association was not valid in Tanner stage 4 girls.

**WHAT IS ALREADY KNOWN ON THIS TOPIC?**

Weight gain has an effect on pubertal development, such as the timing of pubertal initiation and age at menarche.

**WHAT THIS STUDY ADDS?**

In girls with central precocious puberty, increased body mass index affects peak stimulated luteinizing hormone levels during the early pubertal stages (Tanner stages 2 and 3). Excess adiposity may suppress gonadotropin secretion during early puberty through complex hormonal interactions.

## INTRODUCTION

Over the past decades, many studies have reported, in various ethnic groups, an earlier age of onset of puberty and menarche in girls (1,2,3). The timing of puberty is primarily driven by genetic factors. Several genetic mutations have been identified in patients with idiopathic hypothalamic hypogonadism and central precocious puberty (CPP) (4,5). Although genetic factors play a critical role in the timing of puberty, nutrition and environmental factors also influence pubertal development (6). Excess adiposity may be one of the most important causes of alterations in pubertal development, such as the timing of onset of puberty and age of menarche. There are many studies which report the correlation between increasing body mass index (BMI) and early maturation and which also take into account relevant racial and genetic factors (7,8,9,10).

Precocious puberty is characterized by early activation of the pituitary-gonadal axis which leads to increased growth rate and development of secondary sexual characteristics (11). The secretion of gonadotropin-releasing hormone (GnRH) is low during the juvenile period in mammals and increases in amount and in frequency at the onset of puberty (12). So, girls with precocious puberty have high luteinizing hormone (LH) levels and a high LH/follicle-stimulating hormone (FSH) ratio for their age. However, recent studies have shown that the LH increase with the onset of sleep, the earliest hormonal change in puberty, was blunted in otherwise healthy girls with very high BMIs (13,14). In our previous study, high BMI was also associated with lower LH response to the GnRH stimulation test in boys with CPP (15). These findings indicate that obesity affects GnRH secretion and pubertal maturation. Therefore, the purpose of this study was to evaluate LH secretion during GnRH stimulation tests in a subset of normal-weight and obese girls with idiopathic CPP.

## METHODS

The study sample included 981 girls who were diagnosed with idiopathic CPP at Ajou University Hospital between 2008 and 2014. All subjects underwent GnRH stimulation tests as part of their clinical evaluation. Precocious puberty was defined as the appearance of breast development before the age of 8 years, advanced bone age (BA) [one year above chronological age (CA)], and increased LH response to the GnRH stimulation test (peak LH >5 IU/L) on immunoradiometric assay (IRMA) (16). Tanner stage was evaluated by palpation of glandular breast tissue while the subjects raised their arms and evaluations were done by one pediatric endocrinologist. Patients with organic intracranial lesions such as brain tumors, were excluded after neuroradiological examinations using magnetic resonance imaging of the hypothalamic-pituitary region. Subjects with previously identified endocrine disorders, previous use of hormonal medications, those with chromosomal abnormalities, as well as subjects with abnormal androgen secretion and congenital adrenal hyperplasia were excluded from the study. Plasma thyroxin and thyroid-stimulating hormone concentrations were measured in order to exclude hypothyroidism. Ovarian disorders were ruled out on the basis of a pelvic ultrasound. None of the subjects had experienced menarche. The interval between the onset of puberty and the age at diagnosis was 0.60±0.1 year.

The GnRH stimulation test was performed in the daytime. Serum LH and FSH levels were determined at baseline and 30, 45, 60, and 90 min after injection of GnRH (100 µg Relefact; Sanofi-Aventis, Frankfurt, Germany). Basal estradiol (E2) was measured before injection of GnRH. Height was assessed with a Harpenden stadiometer. Weight was measured with a calibrated digital scale. BMI was calculated as weight/height2. Pubertal status (Tanner stage for breast development) was assessed by inspection and palpation and documented by two pediatric endocrinologists. Patients were categorized by pubertal stage (Tanner 2-5) (17). BA was determined using an X-ray of the left hand using the Greulich and Pyle method (18). The standard deviation scores (SDS) for height, weight, and BMI were calculated based on the 2007 Korean National Growth Charts (19).

Serum LH and FSH levels were measured by IRMA (BioSource, Nivelles, Belgium). The detection limits for LH and FSH were 0.1 IU/L and 0.2 IU/L, with an intra-assay coefficient of variation (CV) ranging from 1.4-3.9% to 1.1-2.0% and an inter-assay CV ranging from 3.4-8.0% to 2.4-4.4%, respectively. E2 levels were measured by radioimmunoassay with a detection limit of 5 pg/mL, with an intra-assay CV ranging from 4.0-7.0% and an inter-assay CV ranging from 4.2-8.1% (RIA; Coat-A-Coung, Diagnostic Products, Los Angeles, CA, USA).

### Statistical Analysis

Statistical analysis was performed using SPSS version 21.0 (SAS Institute, Chicago, USA). BMI status was stratified as normal (BMI between the 5th and 85th percentiles), overweight (BMI between the 85th and 95th percentiles), and obese (BMI ≥95th percentile). For comparison of clinical parameters according to BMI, ANOVAs with Tukey’s post-hoc tests were performed for each Tanner stage. Spearman’s correlation was used to examine the relationship between peak LH and clinical parameters according to the Tanner stage because LH levels were not normally distributed. After finding a significant association with peak LH, linear regression was performed in multivariate analysis with stepwise variable selection, including age at diagnosis, BMI SDS, difference between BA and CA, basal LH, basal FSH, and basal E2 levels. Statistical significance was defined as p<0.05. Results are given as mean ± standard deviation unless otherwise stated.

## RESULTS

Mean age at diagnosis of the study group was 8.2±0.86 years. The majority of the children were in Tanner stage 2 (n=610, 62.2%), with 270 (27.5%) children in Tanner stage 3 and 101 (10.3%) in Tanner stage 4 of puberty. Mean BMI SDS was 0.43±0.88 and ranged from -2.39 to 3.16. The numbers of normal-weight, overweight, and obese girls were 733 (74.7%), 169 (17.2%), and 79 (8.1%), respectively. In all Tanner stages, obese girls were significantly taller than normal-weight girls, and BA was more advanced in obese children. As expected, obese children were heavier and had greater BMI values at all Tanner stages (Table 1).

As shown in Table 2, in Tanner stage 2 and 3 girls, the LH response to GnRH stimulation was clearly influenced by BMI status. Peak stimulated LH levels were significantly lower in overweight subjects and lower still in obese subjects. However, peak stimulated LH levels were not significantly different between the groups in Tanner stage 4 girls. There were no differences in basal LH, FSH, or E2 levels in any Tanner stage groups.

On Spearman’s correlation analysis, BMI SDS was significantly and negatively associated with peak LH levels in Tanner stage 2 and 3 girls (r=-0.137, and -0.157; p=0.001 and p=0.010, respectively), while BMI SDS was not significantly associated with peak stimulated LH levels in Tanner stage 4 girls (r=-0.080; p=0.427). Basal LH and BA-CA were significantly associated with peak LH levels (Table 3).

To identify the determinants of peak LH response to the GnRH stimulation test, stepwise multivariate regression analysis was performed. BMI SDS, Tanner stage, basal LH, and BA-CA were significant predictors of peak LH levels (Table 4). BMI SDS was the only negative predictor of peak LH levels.

## DISCUSSION

The present study investigated how obesity affects the LH response to GnRH stimulation test in girls with idiopathic CPP and at different stages of puberty. During the GnRH stimulation test, while peak LH levels were significantly lower in obese and overweight subjects than in normal-weight subjects, in Tanner stage 2 and 3 girls, in girls at later pubertal stages (Tanner stage 4), obesity was not associated with LH secretion. These findings indicate that excess adiposity or fat accumulation may affect gonadotropin secretion in girls at early pubertal stages but not at later pubertal stages.

The beginning of puberty is characterized by marked increases in GnRH and gonadotropin secretion (12). The increased prevalence of overweight and obesity may be triggering early pubertal development (20,21,22). The mechanisms whereby obese children grow faster beginning in early childhood remain unclear. Adipocytes secrete leptin in direct proportion to adipose tissue mass as well as to nutritional status. Leptin and its regulation may be important in the initiation and/or progression of puberty and may play a role in the earlier onset of puberty in obese children compared to normal-weight children (23,24,25). Leptin concentrations are directly correlated with fat mass, and leptin serves as a signal to the hypothalamus regarding energy stores in the adipose tissue compartment (26). Animals lacking functional leptin or its receptor show marked suppression of pulsatile LH secretion and are infertile (27,28). Leptin may also have more direct stimulatory effects on GnRH and gonadotropin secretion (7). So, we hypothesized that obese girls with precocious puberty would have higher levels of LH than normal-weight girls with precocious puberty. However, our results show that excessive adiposity, as assessed by elevated BMI, was correlated with decreased LH secretion in girls with CPP.

There are a small number of reported studies on LH secretion and obesity in children. McCartney et al (14) reported that obesity in prepubertal and early pubertal girls was associated with reduced LH secretion and reduced nocturnal changes in LH compared to their normal-weight counterparts but with increased frequency of LH secretion during later puberty. In another study by Bordini et al (29), spontaneous sleep-related gonadotropin rise was blunted in healthy excessive weight girls undergoing puberty at a normal age. They also reported that peak LH response to a GnRH agonist was not associated with BMI percentile, in contrast to the results of our study. This may be related to the use of a different test agent (GnRH agonist) and the small number of subjects undergoing normal puberty. Recently, Fu et al (30) evaluated peak LH levels in 865 girls with idiopathic CPP. They reported that LH secretion after a GnRH provocation test was lower in overweight and obese girls than in normal-weight girls with CPP, but they did not investigate the differences in LH secretion based on Tanner stage.

The mechanisms by which increased BMI is associated with decreased LH levels are unclear. Sex steroids may directly affect sexual maturation. Estrogen has an essential role in the initiation and progression of puberty, and increased estrogen levels are linked to excess adipose tissue (31). Estrone (E1) and E2 are synthesized from androstenedione and testosterone. Decreased hepatic inactivation by estrogen-2 hydroxylation in the context of obesity leads to reduced estrogen clearance (32). Girls with obesity exhibit increased total testosterone production and reduced hepatic sex hormone binding globulin (SHBG) production, and a decrease in the levels of SHBG could result in increased sex steroid bioavailability (33). Also, increased adiposity could lead to increased aromatase activity, resulting in increased and accelerated conversion of androgens to estrogens (34). Relatively inactive androgens may induce advanced breast development, while the hypothalamic-pituitary axis remains relatively dormant. Another potentiating/mediating factor influencing the effect of BMI on LH is insulin resistance or hyperinsulinemia associated with obesity. Increased fat accumulation leads to increased insulin resistance, which may affect sex hormone production (6). Increased insulin levels in obese girls stimulate androgen production by acting on the adrenal glands, liver, and ovaries. Furthermore, increased androgen levels may affect central neurosecretory function (35).

GnRH secretion is extremely sensitive to negative feedback from sex steroids during early puberty (36). Although our study showed that E2 levels were not significantly different in normal-weight children and obese children in all Tanner stages, lower LH response to GnRH stimulation tests may partly reflect a negative feedback effect by estrogen. Tanner stage 2 and 3 girls may experience relative immaturity of hypothalamic-pituitary function (37). The amount of estrogen required to suppress gonadotropins in peripubertal girls is lower than the amount required in adults (38). Also, the prepubertal hypothalamic-pituitary axis is estimated to be six- to 15-fold more sensitive than that of adults (39). Our study showed that BMI was not significantly associated with peak LH levels in Tanner stage 4 girls. These results suggest that sensitivity to negative feedback decreases as the reproductive axis matures, permitting increasing GnRH and gonadotropin secretion (33).

In our study subjects, obese girls in Tanner stage 2 were significantly younger than those in the other groups. LH levels tend to increase in the later stages of puberty, although FSH levels rise during the early stages of puberty (40). So, early detection of precocious puberty in Tanner stage 2 obese girls may also be the cause of low peak LH levels in the GnRH stimulation test.

This study has a few limitations stemming from its retrospective design. We did not evaluate a variety of other hormones known to link obesity to gonadotropin secretion, such as insulin, SHBG, and testosterone; as a result, we cannot prove causality. Also, our sample size of Tanner stage 4 girls was smaller than that of Tanner stage 2 and 3 subjects. Furthermore, although BMI and peak LH levels were significantly correlated in early pubertal stages, there was a lack of statistical power. Finally, any recruitment bias was especially unlikely to have influenced the results in Tanner stage 2 and 3 girls. Obese girls may present earlier than normal-weight girls because of greater concerns over psychological and health-related body issues. Furthermore, it can be difficult to distinguish lipomastia from true breast tissue in overweight and obese girls. In order to reduce bias, Tanner stage was evaluated by palpation of glandular breast tissue while the subjects raised their arms and evaluations were done by one pediatric endocrinologist. Regardless of these limitations, our findings indicate a potential association between gonadotropin secretion and excess adiposity.

In conclusion, our results suggest that higher BMI during early puberty is associated with slightly lower LH levels evoked by GnRH stimulation in girls with precocious puberty, but increased BMI is not associated with LH secretion in girls with CPP in later pubertal stages. Therefore, BMI should be considered when interpreting the results of GnRH stimulation tests. Further studies are needed to explore the mechanisms by which BMI affects gonadotropin secretion.

## Ethics

Ethics Committee Approval: Retrospective study, Informed Consent: Retrospective study.

Peer-review: External and Internal peer-reviewed.

## Figures and Tables

**Table 1:**
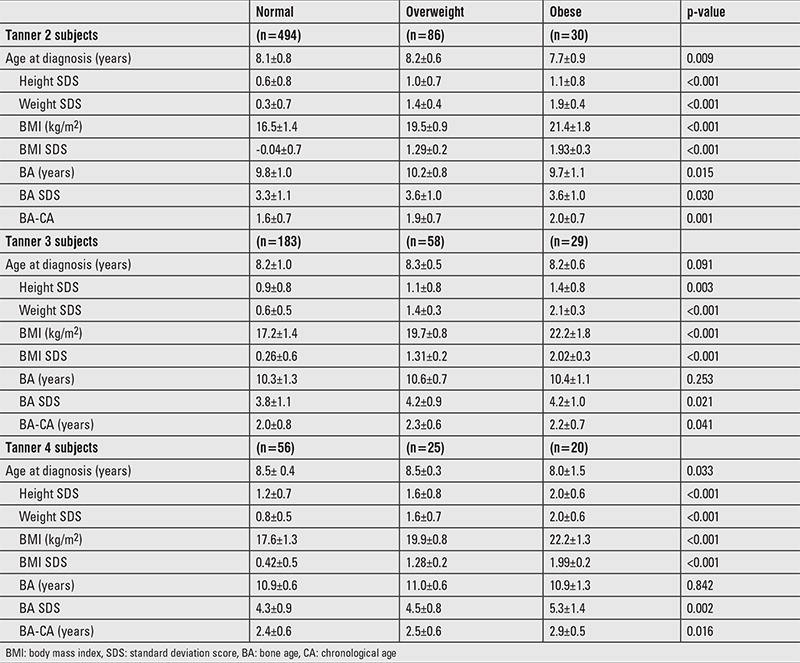
Baseline characteristics of study subjects stratified by body mass index and Tanner stage

**Table 2:**
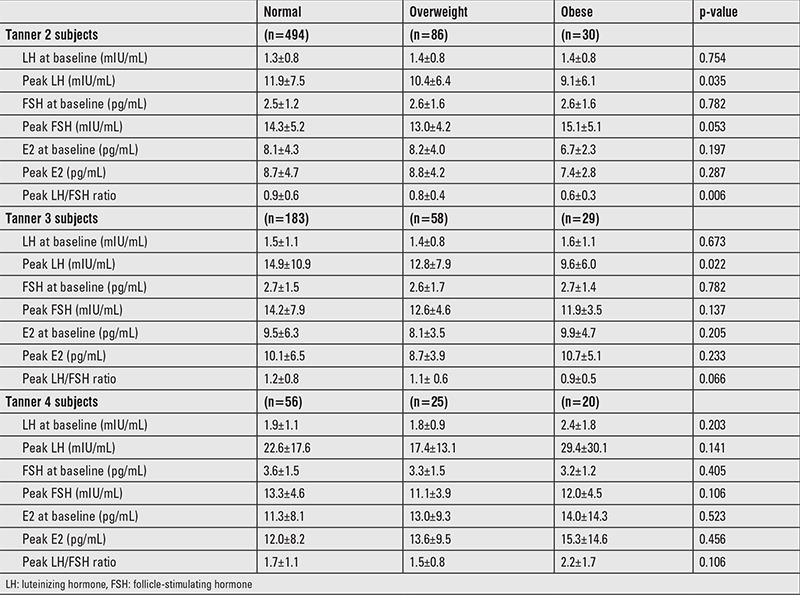
Hormone levels of study subjects stratified by body mass index and Tanner stage

**Table 3:**
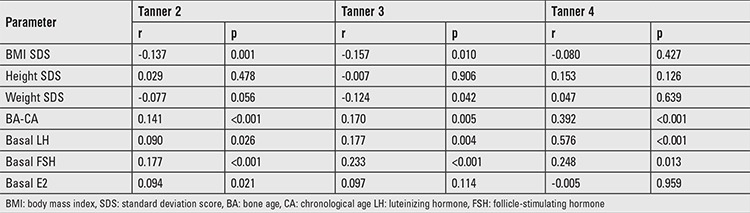
Spearman’s correlation of peak stimulated luteinizing hormone levels with various parameters in all subjects (n=981)

**Table 4:**
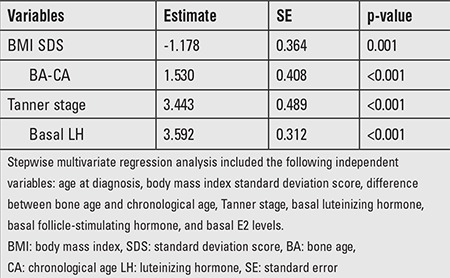
Multivariate analysis of factors associated with peak luteinizing hormone values (n=981, r2=0.220, p<0.001)
